# Genetic Parameters of Effort and Recovery in Sport Horses Assessed with Infrared Thermography

**DOI:** 10.3390/ani11030832

**Published:** 2021-03-16

**Authors:** Ester Bartolomé, Davinia Isabel Perdomo-González, María José Sánchez-Guerrero, Mercedes Valera

**Affiliations:** Departamento de Agronomía, ETSIA, Universidad de Sevilla, Utrera Rd. Km 1, 41013 Sevilla, Spain; daviniapergon@gmail.com (D.I.P.-G.); v32sagum@gmail.com (M.J.S.-G.); mvalera@us.es (M.V.)

**Keywords:** eye caruncle temperature, Spanish Sport Horse, performance test, genetic lines, heritability, infrared thermography

## Abstract

**Simple Summary:**

The way a horse activates (effort phase-EP) and recovers (recovery phase-RP) during a sport event can affect its sport performance. The aim of this manuscript was to test horses’ adaptation to sport performance and its genetic basis, using eye temperature assessed with infrared thermography. EP and RP were measured in 495 Spanish Sport Horses, during a performance test, considering sex (2) and genetic lines (5) as fixed effects. The ranking position obtained on the official sport competition celebrated the day after the performance test was also collected. Differences in variables due to genetic line and sex effects were found, showing that, regardless of the genetic line, stallions tended to recover better than mares after the sport test developed. High positive correlations were found between EP and RP for both fixed effects, so that, the higher the EP, the higher the RP. However, for the ranking position, a low negative correlation was found, so that the higher the eye temperature increase, the better the position. Heritabilities showed medium–high values with a medium positive genetic correlation between them. Thus, breed origins and sex influence horses’ effort and recovery during sport performance, showing a genetic basis adequate for selection.

**Abstract:**

The way a horse activates (effort phase-EP) and recovers (recovery phase-RP) during a sport event can affect its sport performance. The aim of this manuscript was to test horses’ adaptation to sport performance and its genetic basis, using eye temperature assessed with infrared thermography. EP and RP were measured in 495 Spanish Sport Horses, during a performance test, considering sex (2) and genetic lines (5) as fixed effects. The ranking position obtained on an official sport competition was also collected. Differences in variables due to genetic line and sex effects were found, showing that, regardless of the genetic line, stallions tended to recover better than mares after the sport test developed. High positive intra-class correlations (*p* < 0.001) were found between EP and RP for both fixed effects, so that the higher the EP, the higher the RP. However, for the ranking position, a low negative correlation (*p* < 0.01) was found, so that the higher the eye temperature increase, the better the position. Heritabilities showed medium–high values with a medium positive genetic correlation between them. Thus, breed origins and sex influence horses’ effort and recovery during sport performance, showing a genetic basis adequate for selection.

## 1. Introduction

During sport events, horse metabolism produces a large increase in flux of substrate (as glycogen or glucose) to increase fuel availability, maintain acid–base balance within acceptable limits, and limit body temperature. The supply of these substrates is controlled by the hormonal responses to exercise, which include a reduction in blood insulin concentration and increases in blood catecholamine, cortisol, and glucagon concentrations [1]. The increase in catecholamine (also related with a stress response) enhances an increase in heart rate and a splenic contraction, leading into major blood flow into the central circulation [2]. How effective this system is would be an indicator of a horse’s fitness and of a horse’s probability to excel at equestrian events, taking into consideration that the stress response developed depends also on the type of exercise performed [2,3].

Monitoring these physiological changes has been assessed with heart rate (HR) response, blood lactate concentrations, oxygen uptake in relation to exercise intensity [4,5], salivary, hair and blood cortisol [3,6,7,8], or immune cell proliferation [9,10,11]. However, they are all quite difficult measures to be assessed during regular equestrian competitions as riders and owners are unwilling to allow the experimental procedures to impact on the animal and affect their sport results. Furthermore, these methods either require laboratory conditions (oxygen uptake, salivary, and hair cortisol), a blood sample (lactate, blood cortisol, and immune cells), or to touch and distract the horse during the assessment (heart rate).

Lately, some non-invasive tools for physiological changes measurement, such as eye temperature assessed with infrared thermography (IRT) technology, have shown a great potential to assess physiological changes in horses during equestrian competitions and it is also correlated with traditional invasive physiological measures, such as lactate concentration in blood [12,13,14]. This tool can detect small changes in temperature by measuring the radiated electromagnetic energy produced by the horse as a physiological response to exercise. Thus, the increase of catecholamine concentrations, in addition to blood flow responses developed during a sport equestrian event, will produce changes in heat production and heat loss in small areas around the medial posterior palpebral border of the lower eyelid and the lacrimal caruncle. Thus, as both of them have rich capillary beds innervated by the sympathetic system, they respond to changes in blood flow [15].

In general, two physiological phases could be described around a competition event: an “effort phase” (EP), which is the physiological difference between the moment when the animal is calm and the moment when the animal develops the sport performance and reaches the peak of the exercise’s intensity; and a “recovery phase” (RP), comprising the physiological difference from this sport peak to the moment when body homeostasis is completely restored [13].

Furthermore, as occurs with heart rate or lactate levels [16], eye temperature assessed with IRT is not only dependent on the aerobic capacity of the horse, but on inherited parameters, such as breed or age [13,17,18]. This issue takes a greater importance in crossbreeds, such as the Spanish Sport Horse (CDE) horse. The CDE is a recent composite breed formed from crosses with other horse breeds. It was founded in 2002 and the main goal of its breeding program is “to obtain a horse with a good functional conformation, temperament and health, able to attain a high performance at either national or international sports events in which it participates” [19].

Thus, taking into consideration that previous authors [20,21] have found differences between horse breeds due to the physiological adaptation to sports, the physiological response of these CDE crossbred animals during effort and recovery from exercise would be conditioned by the physiological response of the breeds included on their pedigree.

The aims of this study were, first, to test the influence of different environmental effects on effort and recovery by CDE during a performance test, assessed with IRT, and second, to estimate the genetic parameters and genetic correlations between these variables to test their suitability for genetic selection.

## 2. Materials and Methods

### 2.1. Animals

Measurements were obtained from 495 animals (332 stallions and 163 mares) of the Spanish Sport Horse (CDE) breed, aged from 2 to 13 years old. Animals were selected according to their participation in the official show jumping competition celebrated the day after the study. All owners were contacted and informed about the performance test that would be used with their animals. Only those animals whose owners agreed to participate in the study were used for the analyses and were asked to arrive at the competition center one day before the official show jumping competition in order to participate in the performance test. All experiments were performed in accordance with Directive 2010/63/EU guidelines.

According to the composite nature of the CDE, many different breeds were included in the studbook as relatives. In order to consider the influence of the genetic composition on the effort and recovery of the CDE studied, 5 genetic lines were assessed according to their origin. An animal belonged to one of them when the majority of its ancestors was from a breed in this line (genetic contribution ≥ 50%) [12]. In total, 14.6% (72 horses) belonged to a ‘German’ (GE) genetic line (L1), created according to the country of origin of the breeds that conformed it (Holsteiner, Hanoverian, Westphalian, Oldenburger, and Trakehner); 15.2% (75 horses) belonged to a ‘Thoroughbred’ (TH) genetic line (L2); 28.9% (142 horses) belonged to a ‘Trotter’ (TR) genetic line (L3); and 24.9% (124 horses) belonged to a ‘Pura Raza Española’ (PRE) genetic line (L4). In addition, 16.7% (82) of the CDE belonged to a fifth genetic line, referred to as ‘Other Breeds’ (OT; L5), which included those horses with ancestors from other horse breeds and from those CDE with a minority influence (<50%) of the already classified breeds (Table 1).

### 2.2. Study Design

A retrospective cohort study was used with a performance test specifically developed for this study, in order to obtain performance information under the same conditions. The test was held one day before an official show jumping competition that was performed at the same equestrian center, on the competition arena (30 m^2^) with audience in the stands, in order to simulate regular competition environmental conditions. During this specific performance test, the horse developed a 2-min (approximately) routine, beginning with walk, then trot, and followed by gallop movements all around the training arena. Then, the horse finished the performance test by jumping over three simple obstacles 1.00 m, 1.10 m, and 1.25 m high, respectively, all a crossed fence, with an average speed of 250 m/min. All horses analyzed in this study performed the exercise with professional riders (all with Galope 6 or more [22]).

Performance test exercises were held at the same equestrian center, during different show jumping competitions held between 2014 and 2018 in September, hence sharing similar environmental and housing conditions. Temperature ranged from 14 to 24 °C and relative humidity between 40% and 50%. However, for the horses to adapt to the new environmental conditions of the center before the performance test, owners were asked to arrive at the equestrian center at least one day before the performance test (two days before the official show jumping competitions). During their stay, the animals were housed in 3 × 3 m^2^ stall boxes with dry straw as bedding material and were fed with hay, concentrate, and water ad libitum, thus providing standardized environmental and housing conditions.

### 2.3. Physiological Data

The physiological changes of effort and recovery developed by the participating animals during the performance test were assessed with eye temperature (ET) measurements. For this, samples were collected three times per horse during the test day (one day before the official competition): one hour before the performance test (BT) inside the stall box of the animal, just after the test (JAT) within five minutes after the end of the test at the finish line, and one hour after it (AT), when the animal was resting on the stall box. For the infrared photographs collection, all horses were handled by the owner or their regular horse keeper on the place of the collection (stall boxes for BT and AT measurements and the entrance to the performance arena for JAT measurements). The camera operator was placed 1 m away from the horse, perpendicular to its left eye where the images were taken without touching the horse. In order for the horses to get used to the operators and to the camera itself, a short period of habituation was carried out before the test day with all the horses. During this habituation period, the horse could freely sniff the camera and the camera operator.

To obtain effort and recovery eye temperature measurements, differences between phases were calculated. Thus, the eye temperature of the ‘effort phase’ (EP) was computed as the difference between JAT and BT eye temperature measurements, whereas the eye temperature of the ‘recovery phase’ (RP) was computed as the difference between JAT and AT measurements. Hence, for the purposes of this study, statistical analyses were developed just for EP and RP measurements.

**Figure 1:**
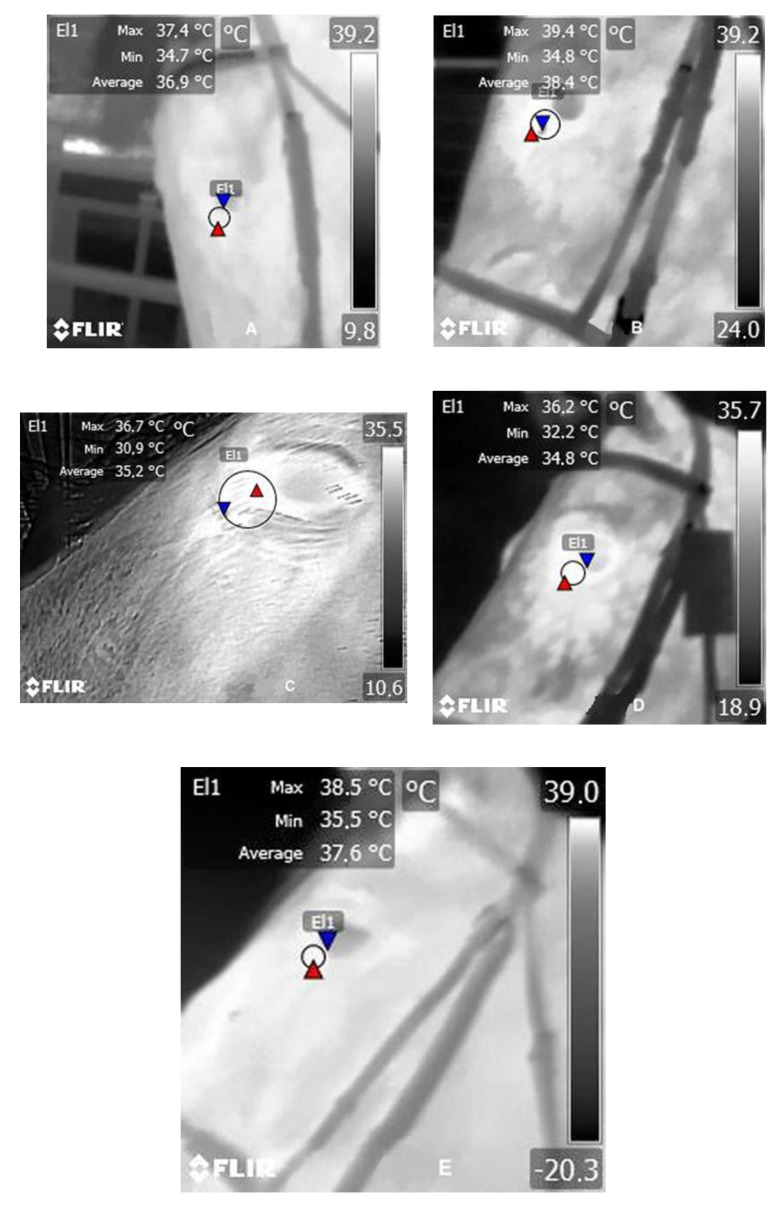
Analyzed eye temperature images of CDE horses according to their genetic line, obtained just after the performance test. (**A**) German genetic line L1; (**B**) Thoroughbred genetic line L2; (**C**) Trotter genetic line L3; (**D**) Pura Raza Española genetic line L4; (**E**) Other Breeds genetic line L5. Where, **El1** is a selected area from the thermographic image (indicated with a circle); 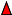 indicates the maximum temperature point; and 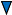 indicates the minimum temperature point.

Eye temperature images were taken always by the same person, with a portable infrared thermography (IRT) camera (FLIR E60. FLIR Systems AB, Danderyd, Sweden). In order to calibrate the camera results, environmental temperature and relative humidity were recorded with a digital thermohygrometer (Extech 44550, Extech Instruments, Nashua, New Hampshire) every time an eye temperature sample was taken. To determine eye temperature, an image analysis software FLIR Tools 6.0.17046.1002 (FLIR Systems AB, Danderyd, Sweden) was used, measuring the temperature (°C) within an oval area traced around the caruncle of the eye, including approximately 1 cm around it. The program provided the maximum, minimum, and mean temperature of the oval area previously traced (Figure 1), but for the study purposes, only the maximum temperature was used. Three-four images were taken per animal and collection period. Later, the best image was selected to obtain the data for the study.

### 2.4. Statistical Analyses

A previous Shapiro–Wilk test (results not shown) presented a normal distribution of the variables studied. Hence, parametric statistical analyses and a Bayesian approach for the genetic parameters were used.

In order to test the influence of horse characteristics on the physiological adaptation of the studied horses, a general linear model (GLM) analysis was developed for the EP and RP variables measured, considering the age as a covariate and sex and genetic line as fixed effects, resulting in the two of them being statistically significant for both parameters (*p* < 0.05; results not shown). According to these results, a least square means analysis was used due to the sex and genetic line for EP and RP variables. Moreover, to determine statistically significant (*p* < 0.05) differences between fixed effects’ levels, a post-hoc Duncan’s test was developed.

To obtain the phenotypic correlations between EP and RP variables, direct and intra-class Pearson’s correlations were computed, according to the genetic line and sex effects. Furthermore, in order to analyze the relation of these variables, with the sport performance developed in the official show jumping competitions held the day after the performance test, direct and intra-class Pearson’s correlations were computed between the ranking position obtained by the animals on the competition (RANK), and both EP and RP variables obtained on the performance test, according to genetic line and sex effects.

For the statistical analyses, the Statistica software v. 8.0 (Stat Soft. Inc., Tulsa, OK, USA) was used.

### 2.5. Genetic Model and Genetic Parameters’ Estimation

The pedigree information was gathered from the studbook provided by the National Association of Spanish Sport Horse Breeders (ANCADES). The pedigree matrix was built 4 generations up with the known pedigree of the analyzed horses, to a total of 7907 animals (3000 males and 4907 females). A BLUP genetic evaluation was computed based on a bivariate animal model using a Bayesian approach. The equation in matrix notation to solve the mixed model was:

$$y\;=\;Xb\;+\;Zu\;+\;e,\;\mathrm{with}\;\left(\begin{array}{l} u\\ e \end{array}\right)∼N\left(\left[\begin{array}{l} 0\\ 0 \end{array}\right],\left[\begin{array}{cc} A\sigma_{u}^{2} & 0\\ 0 & I\sigma_{e}^{2} \end{array}\right]\right),$$

where y is the vector of observations, X is the incidence matrix of systematic effects, Z is the incidence matrix of animal genetic effects, b is the vector of systematic effects, u is the vector of direct animal genetic effects, e is the vector of residuals, $\sigma_{u}^{2}$ is the direct genetic variance, $\sigma_{e}^{2}$ is the residual variance, I is an identity matrix, and A is the numerator relationship matrix.

The fitted model included EP and RP as continuous variables, age as a covariate, two fixed effects: sex (2 levels) and genetic group (5 levels), and two random effects: rider (182 levels) and the rider–horse interaction (275 levels).

As regards to the genetic evaluation computed, phenotypic variance, heritability of the animal, the rider, the rider–horse interaction, and the residual effect for both EP and RP variables and genetic correlations between them were estimated with TM software [23].

### 2.6. Ethic Statement

No specific ethical approval was required for this study due to the performance test being just warm-up exercises that the animals performed regularly before official competitions, for which we did not have to develop any specific protocol or to use specific animals. As regards to temperature samples obtained from the animals, since the eye temperature assessed with infrared thermography was a non-invasive physiological measure collected from a minimum distance of 0.5–1 m away from the animal, it was not necessary to obtain a specific permit for animal experimentation since no pain nor physical stress was afflicted on the animal. Likewise, all the owners of the animals were previously informed of the entire procedure and the type of samples to be taken, obtaining the approval of all the owners of the animals participating in the study.

## 3. Results

### 3.1. Influence of Sex and Genetic Line of Effort and Recovery 

**Figure 2:**
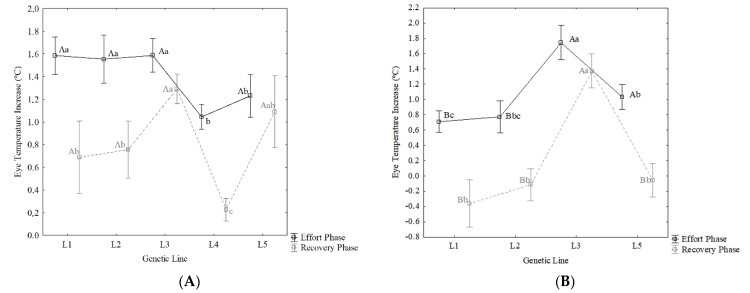
Least square means analysis (means ± standard deviation) according to sex and genetic line and post-hoc Duncan’s test between means. (**A**) Stallions and (**B**) mares. Where L1 is the German genetic line; L2 is the Thoroughbred genetic line; L3 is the Trotter genetic line; L4 is the Pura Raza Española genetic line; and L5 is the Other Breeds genetic line. Different capital letters indicate statistically significant differences (*p* < 0.05) between sexes and within variables, whereas different lowercase letters indicate statistically significant differences (*p* < 0.05) between genetic lines and within variables.

A least square means analysis was computed for EP and RP variables (Figure 2), indicating statistically significant (*p* < 0.05) differences between the means for the genetic line and sex effects.

First of all, it has to be noticed that no mares were registered for L4 CDE horses (PRE genetic line), thus Figure 2B shows results just for the L1, L2, L3, and L5 CDE genetic lines. As regards to the EP variable, L1 and L2 stallions (PRE and TH genetic lines, respectively) showed the highest ET increases (1.59 and 1.57, respectively) and differed significantly to L1 and L2 mares, which showed lower effort increases (0.70 and 0.71, respectively), indicating lower physiological differences from the rest to performance moment. As regards the RP variable, L1, L2, and L5 mares (GE, TH, and OT genetic lines, respectively) showed values below 0 (−0.39, −0.18, and −0.04, respectively), indicating a bad physiological recovery of the mares from those genetic lines, from the exercise and the animal’s need for longer recovery periods. On the other hand, L3 stallions and mares (TR genetic line) showed the greatest statistically significant recovery from the exercise, with 1.28 and 1.60, respectively.

### 3.2. Phenotypic Correlations between Effort and Recovery

Phenotypic Pearson’s correlations (Table 2) between RP and EP revealed a medium positive and statistically significant (*p* < 0.001) correlation (+0.53).

According to genetic line and sex intra-class correlations, positive and statistically significant (*p* < 0.001) correlations were found for L1, L2, L3, and L4 genetic lines and both sexes, ranging from +0.41 for L4 (PRE genetic line) to +0.82 for L1 (GE genetic line) and from +0.46 for stallions to +0.65 for mares. On the other hand, the intra-class Pearson’s correlations between EP and RP variables with RANK position showed negative and statistically significant correlations (*p* < 0.01) between RANK and both EP and RP variables (−0.16 and −0.14, respectively). Finally, intra-class negative and statistically significant correlations (*p* < 0.01) were found with mares (−0.34) and L1 and L2 genetic lines (−0.46 and −0.45, respectively) for EP and just for mares (−0.32) for RP variables. These negative correlations indicated that the higher the temperature increase obtained between exercise and rest (hence, the higher the effort response), the lower the RANK position obtained by the animal on the show jumping competition developed the day after the performance test, thus the best classification.

### 3.3. Genetic Parameters

Finally, the genetic parameters for both physiological variables (EP and RP) are shown in Table 3. The heritabilities obtained for EP (0.26) and RP (0.52) showed a medium to high genetic basis, respectively, for these physiological variables. On the other hand, the ratios for the rider–horse interaction effect (0.37 and 0.33 for EP and RP, respectively) were higher than the ratios for the rider effect (0.15 for both parameters), indicating a greater genetic basis for the interaction than for the rider effect alone. Lastly, the genetic correlation computed between EP and RP (+0.23) indicated a moderate and positive genetic relation between them.

## 4. Discussion

Previous studies have reported that the physiological response of a horse during a sport event is influenced by several factors (sex, breed, environment, rider, fitness of the horse) that would determine its magnitude [1,24,25]. Some of these factors are genetically determined, such as sex or breed. Thus, the knowledge of the magnitude of this response (already settled in the horse from its birth) could be an appropriate tool for the CDE breeding program, as it could help in planning the horses to be mated to obtain animals that could adapt better to the sport competitions they are trained for. Previous studies have also reported differences in the physiological response during competitions due to the horse breed [21,26] that could comprise differences in adapting to new stimuli. These breed differences become key to the selection plans for a composite breed like the CDE, with different breeding recommendations regarding the predominant breed on the CDE analyzed.

Firstly, our results showed that CDE with more than 50% of Trotter breeding in their pedigree (L3) seem to show a greater physiological response before exercise than CDE with other breeds on their pedigree. However, these animals also showed the highest recovery after the exercise, being the fastest to recover their internal homeostasis. This is in accordance with previous studies that found Trotter horses as being selected for speed sport performance (trotting races) and hence, indirectly adapted to become physiologically activated and recover very fast during the performance [27]. Previous studies in humans supported this hypothesis, showing that well-conditioned athletes have shorter recovery times and take longer to become physically fatigued than less fit individuals [28]. 

Instead, those CDE with more than 50% of PRE (L4) on their pedigree showed the least effort values during this test with a long recovery. This could be due to the PRE influence, which gives calmer behavioral attributes [18], that would be expected to develop a small physiological response between phases, and hence, a small EP and RP phases. 

As regards to CDE differences due to sex, a better physical condition would be expected in stallions, due to the more athletic predisposition in males than in females [29]. However, [30] reported a greater proportion of fatal humeral fracture in TH males than in females during races, thus expecting a higher physical level due to a greater effort for the first. This would be in accordance with our results, which showed lower EP and RP results for mares regardless of their genetic line. 

As regards the estimation of the genetic parameters for EP and RP variables, the moderate positive genetic correlation found between variables indicated a good potential to include both in the CDE breeding program, either as a selection criterion or as a fixed effect in the genetic model, as it indicates that, despite there being some relation, they can be selected independently. Thus, this breed could be selected due to physiological parameters that would be an indicator of the welfare state of the animal. On the other hand, these results were slightly higher than those reported by [18] in young PRE horses during dressage competitions, probably due to the higher number of animals studied in this paper, which would lead to better genetic estimation of the data. However, these results were in accordance with the results from [31], with heritability values ranging between 0.2 and 0.5 for different behavioral traits (reactivity reactions and learning abilities), which would certainly condition the magnitude of the physiological response of the animal during exercise.

High and statistically significant phenotypic intraclass correlations were found between most genetic lines (L1 to L4) and both sexes denoted that there was a tendency within breeds and sexes to react and recover from the exercise with a similar magnitude. Hence, CDE with genealogical influence from breeds that tended to develop a big physiological response to exercise tend to develop a fast recovery, like the L3 (TR) and L2 (TH) genetic lines. On the other hand, those with a genetic influence from breeds that tended to show a small physiological response to exercise will be expected to show a long recovery from it, like the L4 (PRE) and L1 (GE) genetic lines. Previous studies about differences in horse breeds due to physiological reactions support our results, with PRE and GE breeds showing more proactive and calm responses compared to TR and TH breeds, which are selected for explosive performances that would demand more reactive and temperamental animals [21,26]. Furthermore, [32] reported differences in digital tendons’ predisposition to injury in sport horses due to the breed, with Warmblood and TH horses failing more at a higher strain and load than Friesian horses. This supports our findings, with differences in mechanical sport properties conditioning also resulting in differences in physiological response to exercise, due to horse breed.

In order to ascertain how the physiological response developed during effort and recovery phases could affect the sport performance of these CDE horses, EP and RP values were correlated with the ranking obtained by the animals the day after the test, in a regular show jumping competition. Our results indicated a low to medium relation with both parameters, so that the higher the physiological response in the EP and RP phases, the better the ranking position. This is in accordance with previous studies in sport horses that found that the best show jumping individuals were the most reactive ones [11,21]. As regards to intra-class correlations due to sex and genetic line, it appeared that only mares for both variables and L1 and L2 CDE (GE and TH genetic lines, respectively) for EP were influenced by the magnitude of their physiological response when competing. This could be due to the temperament, mechanical sport properties, and reactivity differences between breeds and sexes reported previously [26,32], which could bias the sport results of these animals. Otherwise, these results were considered exploratory, and it must be considered that type I errors were used, thus determining the results highlighted in this study. However, before these can be considered as new selection criteria, more research is required, including these variables as a regular measurement on the equestrian events. Furthermore, large studies carried out over several years and containing more animals are needed before any precise measures concerning the influence of the genetic and environmental effects can be determined.

## 5. Conclusions

The results obtained in this study indicated that eye temperature assessed with IRT appears as an adequate tool to evaluate the physiological effort and recovery developed by CDE during exercise and thus, its fitness. Heritability and genetic correlations obtained for EP and RP variables indicated an adequate potential to be included in the CDE breeding program. However, more research is required to confirm the results found here.

## Figures and Tables

**Table 1:** Description of the Spanish Sport Horse (CDE) genetic lines.

Genetic Line	Name	Description	N (%)
L1	German	More than 50% of the CDE ancestors belonged to German horse breeds: Holsteiner, Hanoverian, Westphalian, Oldenburger, or Trakehner.	72 (14.6%)
L2	Thoroughbred	More than 50% of the CDE ancestors belonged to Thoroughbred breed.	75 (15.2%)
L3	Trotter	More than 50% of the CDE ancestors belonged to trotter horse breeds	142 (28.9%)
L4	Pura Raza Española	More than 50% of the CDE ancestors belonged to Pura Raza Española breed	124 (24.9%)
L5	Other Breeds	Included CDE horses with more than 50% of their ancestors from other sport horse breeds (KWPN, Zangersheide, etc.) and CDE horses with less than 50% of their ancestors from the already classified breeds (TR, PRE, TH or GE).	82 (16.7%)

**Table 2:** Direct and intra-class Pearson’s correlations (±standard error) according to sex and genetic line effects, between effort phase (EP) and recovery phase (RP) and the ranking position (RANK), where S refers to the stallions, M to the mares, L1 is the German genetic line, L2 is the Thoroughbred genetic line, L3 is the Trotter genetic line, L4 is the Pura Raza Española genetic line, and L5 is the Other Breeds genetic line. * *p* < 0.05; n.s. not statistically significant.

	EP			RP		
	Direct	Intra-Class		Direct	Intra-Class	
		Sex	Genetic Line		Sex	Genetic Line
RANK	−0.16 (±0.044) *	S: −0.10 (±0.045) ^n.s.^ M: −0.34 (±0.042) *	L1: −0.46 (±0.040) * L2: −0.45 (±0.040) * L3: −0.11 (±0.045) ^n.s.^ L4: −0.02 (±0.045) ^n.s.^ L5: −0.13 (±0.045) ^n.s.^	−0.14 (±0.045) *	S: 0.06 (±0.045) ^n.s.^ M: −0.32 (±0.043) *	L1: −0.29 (±0.043) ^n.s.^ L2: −0.05 (±0.045) ^n.s.^ L3: −0.01 (±0.045) ^n.s.^ L4: −0.08 (±0.045) ^n.s.^ L5: −0.05 (±0.045) ^n.s.^
RP	0.53 (±0.38) *	S: 0.46 (±0.040) * M: 0.65 (±0.034) *	L1: 0.82 (±0.026) * L2: 0.63 (±0.035) * L3: 0.53 (±0.038) * L4: 0.41 (±0.041) * L5: 0.28 (±0.043) ^n.s.^			

**Table 3:** Phenotypic variance, mean, and standard deviation of the marginal posterior distributions for the heritabilities of horse, rider, rider–horse interaction, residual effects, and genetic correlation (*rg*) between both eye temperature variables analyzed, where EP is effort phase, RP is recovery phase, Vp is phenotypic variance, h^2^ is animal heritability, Vr is variance of rider effect/phenotypic variance, Vrh is variance of rider–horse interaction effect/phenotypic variance, Vres is variance of residual effect/phenotypic variance, and s.d. is standard deviation.

	Vp	h^2^ (±s.d.)	Vr (±s.d.)	Vrh (±s.d.)	Vres (±s.d.)	*r_g_*
EP	1.010	0.26 ± 0.158	0.15 ± 0.096	0.37 ± 0.203	0.21 ± 0.096	0.232
RP	0.918	0.52 ± 0.073	0.15 ± 0.057	0.33 ± 0.080	0.01 ± 0.003	
